# Social Context of Adherence in an Open-Label 1 % Tenofovir Gel Trial: Gender Dynamics and Disclosure in KwaZulu-Natal, South Africa

**DOI:** 10.1007/s10461-016-1339-4

**Published:** 2016-03-05

**Authors:** Kathleen M. MacQueen, Sarah Dlamini, Brian Perry, Eunice Okumu, Steve Sortijas, Chitra Singh, Diantha Pillay, Alesha Majors, Sonja Jerome, Sharon Watson, Salim Abdool Karim, Quarraisha Abdool Karim, Leila E. Mansoor

**Affiliations:** 1 Global Health Research, FHI 360, 359 Blackwell Street, Suite 200, Durham, NC 27701 USA; 2 CAPRISA, University of KwaZulu-Natal, Durban, South Africa

**Keywords:** Microbicide, Tenofovir gel, Adherence, Gender dynamics, Disclosure

## Abstract

CAPRISA 008, an open-label extension study of tenofovir gel with coitally-related dosing, provided an opportunity to explore the relationship between product adherence and gender dynamics in a context where women knew they were receiving an active product with evidence of HIV prevention effectiveness. Interviews with 63 CAPRISA 008 participants and 13 male partners in KwaZulu-Natal, South Africa, highlighted that the process of negotiating gel use was determined in part by relationship dynamics including the duration of the relationship, the living situation, an evaluation of the relationship (e.g., partner intimacy and relationship expectations) and culturally-defined steps for formalizing the relationship. While disclosure facilitated adherence for many, others reported using the gel effectively with no disclosure, and in some situations disclosure was a barrier to adherence. Women should be supported in their choice about what to disclose and have opportunity to use this and similar products without their partners’ knowledge or acquiescence.

## Resumen

CAPRISA 008, un estudio de etiqueta abierta de extensión del gel de tenofovir con la dosificación relacionada al coito, que brindó la oportunidad de explorar la relación entre la adherencia del producto y la dinámica de género en un contexto donde las mujeres sabían que estaban recibiendo un producto activo con eficacia evidenciada en la prevención del VIH. Las entrevistas con las participantes de 63 CAPRISA 008 y 13 parejas masculinas en KwaZulu-Natal, Sudáfrica, resaltaron que el proceso de negociación de la utilización del gel fue determinado en parte por la dinámica de la relación, incluyendo la duración de la relación, la situación de vivienda, una evaluación de la relación (por ejemplo, la intimidad de la pareja y las expectativas de la relación) y pasos definidos culturalmente para formalizar la relación. Si bien la divulgación del uso del producto facilitó la adherencia para muchas, otras informaron que usaron el gel efectivamente sin importar la divulgación del uso, y en algunas situaciones divulgación de datos fue una barrera a la adherencia. Las mujeres deben ser apoyadas en su decisión sobre divulgación y tener la oportunidad de utilizar este y otros productos similares sin el consentimiento de su pareja.

## Introduction

An increasingly broad range of HIV prevention products containing antiretrovirals (ARVs) are being evaluated in clinical trials including pills, vaginal and rectal gels, vaginal rings, and long-acting injectables. One of the most consistent findings from HIV prevention trials of products requiring behavioral adherence is that product adherence by women may be undermined or supported as a result of gender dynamics including norms surrounding sexual negotiation, culturally defined gender roles, gender-based violence, and economic vulnerability [1].

There are limited data on the extent to which findings about product disclosure to male partners and adherence by women during a randomized, blinded clinical trial is generalizable beyond the trial context [2]. Women need to explain not only the product being tested but the fact that they do not know if it will work, that they may be using a placebo product, and that the risks of product use may also not be fully known. Disclosure to male partners and the extent to which partners are willing to allow or support product use are likely to be complicated by these factors [3]. Two trials of tenofovir gel in KwaZulu-Natal, South Africa—one a randomized, blinded, placebo-controlled trial (CAPRISA 004) [4] and the other an open-label implementation trial (CAPRISA 008) [5]—provided an opportunity to explore gender dynamics and disclosure in more depth.

Disclosure was shown to have a statistically significant but moderate relationship with tenofovir gel adherence in the CAPRISA 004 trial, and no relationship with estimated effectiveness of the gel [6]. In-depth interviews conducted with women participating in the trial indicated that among those who disclosed gel use to at least one partner, few reported gel use difficulties and most said they received supportive or neutral reactions from their partners [7]. Many said they were comfortable inserting gel in the presence of a partner, that partners may facilitate or provide reminders, and that they were able to use gel for unexpected partner visits. In contrast, among women who said they did not disclose gel use to any partners, about one-third reported gel use difficulties. They were unable to use gel when the partner was present, found it difficult to use gel for unexpected partner visits, expressed concern that a partner may feel cold or wetness from the gel, and some said it was difficult to hide gel from a partner. A small number of those who did not disclose said they were afraid to do so because a partner may be angry or leave, may not want her to use the gel, or may no longer want to use condoms.

Two years following the report of findings of effectiveness from the CAPRISA 004 tenofovir gel trial in South Africa, HIV-uninfected women participating in that trial were invited to participate in an open-label study comparing tenofovir gel delivery using a 2–3 monthly family planning model of delivery with monthly gel delivery per the original trial design. The follow-on study, referred to as CAPRISA 008, provided an opportunity to look at gender dynamics in a context where women knew they were receiving an active product with demonstrated effectiveness. Here we report on qualitative findings from an ancillary study (CAPRISA 106) conducted while the CAPRISA 008 trial was being implemented to provide data on the social acceptability of coitally-related use of tenofovir gel among women and men; the influence of gender dynamics on tenofovir gel use disclosure; and the social barriers and facilitators associated with tenofovir gel use. This study is unique in providing insight into the way adherence factors reflect product attributes and dosing rather than the placebo-controlled clinical trial context. This is the only study reporting how women negotiate use of a microbicide gel in the context of an open-label study.

## Methods

The study was approved by the University of KwaZulu-Natal’s Biomedical Research Ethics Committee in Durban, South Africa and FHI 360’s Protection of Human Subjects Committee in Durham, North Carolina, USA. Written informed consent was obtained from all participants in the language of their preference (isiZulu or English).

### Recruitment

The research took place in KwaZulu-Natal, South Africa, and included women and men aged 18 years and older in the rural Vulindlela subdistrict and urban eThekwini municipality where the CAPRISA 008 implementation trial was underway. Women were eligible if they were actively participating in the CAPRISA 008 implementation trial and not currently taken off study product (e.g., due to pregnancy); all were sexually active, HIV-uninfected, and non-pregnant at the time of enrollment into CAPRISA 008. CAPRISA 008 participants were screened for eligibility into CAPRISA 106 during CAPRISA 008 study visits. A screening tool was used to (1) assess their interest in learning more about CAPRISA 106; (2) find out whether or not they fully disclosed tenofovir gel use to at least one of their partners; and (3) if they had disclosed, if they were willing to refer their partner. To ensure adequate representation of the range of disclosure experiences in the study, women were purposively recruited based on disclosure status. Men were eligible if they were referred by a CAPRISA 008 participant enrolled in this study.

We recognized that the recruitment of male partners of CAPRISA 008 participants could introduce the potential for harm if a woman had not fully disclosed all aspects of study participation and tenofovir gel use to her partner prior to him being interviewed. To protect against such social harm, we screened women for the extent of disclosure with their male partner prior to asking if she would be willing to have her partner recruited for the study. Further, all male partners were recruited through the CAPRISA 008 participant; no male partners were approached for recruitment directly. In order for a male partner to be recruited, a woman had to confirm that she had disclosed (a) that she was participating in the CAPRISA 008 study, (b) that as a participant she was using a vaginal gel designed to reduce her risk of HIV infection, and (c) that the gel contained an ARV product. Women who answered yes to all three criteria were classified as “full disclosers” for the purpose of this study.

### Data Collection

The in-depth interview (IDI) data collection instruments were piloted in isiZulu. No implementation issues were identified during piloting that resulted in significant changes to the instruments; therefore, the pilot IDI data were included as part of the final data analysis. To enhance participant comfort levels, IDIs were conducted by study staff of the same sex as the participant. IDI participants had the option to be interviewed at a mutually agreed upon location in the community including the CAPRISA research clinics; non-clinic locations were used if confidentiality and staff safety could be assured. The IDIs focused on exploring the social dimensions of tenofovir gel adherence and continuation, and were conducted individually with women and their male partners.

Data collection was documented through digital audio recordings supplemented by expanded notes from the field team summarizing emergent issues and overall quality of the interaction with participants. Each recorded IDI was first transcribed in isiZulu and then translated to English, following a transcription protocol. The f4 transcription program (Audiotranskription, Marburg, Germany) was used to transcribe the isiZulu IDIs verbatim from the audio recordings by CAPRISA field staff. Following transcription they went through a quality control check process of the original audio after which the transcripts were translated from IsiZulu to English. A quality check of the English translation of the transcript was conducted to ensure the content reflected what was discussed during data collection.

English versions of the transcripts included notes on cultural practices as needed to ensure appropriate interpretation of meaning during coding. These notes were written by field team members who were themselves Zulu. During one interview with a male partner of a woman enrolled in CAPRISA 008, the participant refused to be audio recorded; therefore, the interviewer took detailed notes during the interview regarding the participant’s response to each section of the interview guide and then expanded his notes with further detail immediately after the interview was completed.

Since adherence was the primary outcome for CAPRISA 008, and data were collected while the trial was still underway, women were interviewed without knowledge of adherence as measured in the trial nor did we collect self-reported data on gel adherence.

### Data Analysis

Qualitative data analysis was done using a combination of structural coding to identify text associated with specific topics of inquiry covered in the interview guide, thematic analysis to identify broad emergent themes [8], and constant comparison [9] to drill down into specific topics and themes for a detailed analysis. Analyses were conducted using NVivo 10 (QSR International Pty Ltd, Doncaster, Victoria, Australia). For the thematic analysis a team of five analysts independently reviewed a sample of 15 transcripts from across the participant groups, identified broad themes, and developed an initial codebook including definitions and examples of the emergent topics comprising each theme. Three analysts then coded all transcripts and expanded notes using an iterative process to refine the codebook, ensure all salient text was coded and corroborate individual interpretations of the data. Approximately 10 % of the transcripts were preselected to assess inter-coder reliability. The three analysts independently coded the same transcripts and any discrepancies in interpretation of the data or in application of the codes were identified and resolved, and transcripts recoded as needed.

Once thematic coding was completed, all coded text related to the following themes was abstracted for the constant comparison analysis reported here: perceptions of gel, relationship dynamics, gel adherence methods and disclosure of gel use/study participation. The abstracted text for each of these broad themes was analyzed for emergent content by a single analyst using constant comparison; coding and codebook development was iterative and inductive. When the abstracted text for each theme was fully coded each analyst generated summary frequency tables highlighting the emergent content. Another analyst then independently reviewed the summary tables and coded text to confirm the results. Any discrepancies in interpretation of the data or frequency of main themes were discussed until agreement on each of the results was reached.

## Results

A total of 63 women were interviewed (34 urban, 29 rural) and 13 male partners (5 urban, 8 rural). Table 1 provides sociodemographic information on the 63 women participants, derived from CAPRISA 008 baseline data. The four main themes explored in this analysis (perceptions of gel, relationship dynamics, gel adherence methods, and disclosure of gel use/study participation) were discussed in each of the IDIs from both research sites.

**Table 1:** Characteristics of CAPRISA 008 participants included in the CAPRISA 106 study, by site and disclosure status (derived from CAPRISA 008 baseline data)

	Rural (n = 29) % (n)				Urban (n = 34) % (n)				Overall (n = 63) % (n)
	Disclosed (n = 17)	Partially disclosed (n = 5)	Non-disclosed (n = 7)	Overall (n = 29)	Disclosed (n = 20)	Partially disclosed (n = 11)	Non-disclosed (n = 3)	Overall (n = 34)	
Age group (years)									
18–25	35 (6)	0 (0)	57 (4)	34 (10)	15 (3)	36 (4)	33 (1)	24 (8)	29 (18)
26–35	41 (7)	60 (3)	43 (3)	45 (13)	55 (11)	64 (7)	67 (2)	59 (20)	52 (33)
36 or older	24 (4)	40 (2)	0 (0)	21 (6)	30 (6)	0 (0)	0 (0)	18 (6)	19 (12)
Highest level of education completed									
Grade 8 or less	12 (2)	20 (1)	0 (0)	10 (3)	0 (0)	0 (0)	0 (0)	0 (0)	5 (3)
Grade 9	0 (0)	20 (1)	0 (0)	3 (1)	0 (0)	0 (0)	0 (0)	0 (0)	2 (1)
Grade 10	24 (4)	20 (1)	14 (1)	21 (6)	15 (3)	0 (0)	0 (0)	9 (3)	14 (9)
Grade 11	24 (4)	20 (1)	43 (3)	28 (8)	20 (4)	9 (1)	33 (1)	18 (6)	22 (14)
Grade 12	29 (5)	20 (1)	29 (2)	28 (8)	50 (10)	82 (9)	67 (2)	62 (21)	46 (29)
Completion of tertiary education	12 (2)	0 (0)	14 (1)	10 (3)	15 (3)	9 (1)	0 (0)	12 (4)	11 (7)
Age of primary partner (years)									
18–25	12 (2)	0 (0)	0 (0)	7 (2)	5 (1)	0 (0)	0 (0)	3 (1)	5 (3)
26–35	53 (9)	60 (3)	100 (7)	66 (19)	60 (12)	82 (9)	100 (3)	71 (24)	68 (43)
36 or older	35 (6)	40 (2)	0 (0)	28 (8)	35 (7)	18 (2)	0 (0)	26 (9)	27 (17)
Average difference in age between participant and primary partner (years)^a^	4.2	2.4	4.0	3.9	4.0	5.7	3.7	4.5	4.2
HIV status of primary partner									
Negative	65 (11)	60 (3)	86 (6)	69 (20)	55 (11)	55 (6)	100 (3)	59 (20)	63 (40)
Positive	0 (0)	0 (0)	0 (0)	0 (0)	5 (1)	0 (0)	0 (0)	3 (1)	2 (1)
Unknown	35 (6)	40 (2)	14 (1)	31 (9)	40 (8)	45 (5)	0 (0)	38 (13)	35 (22)

^a^Absolute value; includes 3 women who were older than their partner [one rural (difference of 2 years) and two urban (differences of 2 and 4 years)]

Analysis of thematic content revealed few differences by site. When comparing rural and urban female IDI participants, rural women more than urban women discussed community perceptions that CAPRISA was a center for people with HIV infection (rural 18/29 = 62 %; urban 8/34 = 24 %), mentioned suspiciousness towards research more often (rural 11/29 = 38 %; urban 8/34 = 24 %), and more frequently reported community perceptions that the gel causes HIV (rural 15/29 = 52 %; urban 7/34 = 21 %). Urban women indicated greater uncertainty about the gel’s effectiveness in preventing HIV in women compared with their rural counterparts (urban 13/34 = 38 %; rural 4/29 = 14 %) and more frequently mentioned the gel’s partial ability to protect women (urban 9/34 = 26 %; rural 2/29 = 7 %). Unless otherwise noted, results are reported for the urban and rural sites combined, given these minimal differences in thematic content.

**Fig. 1:**
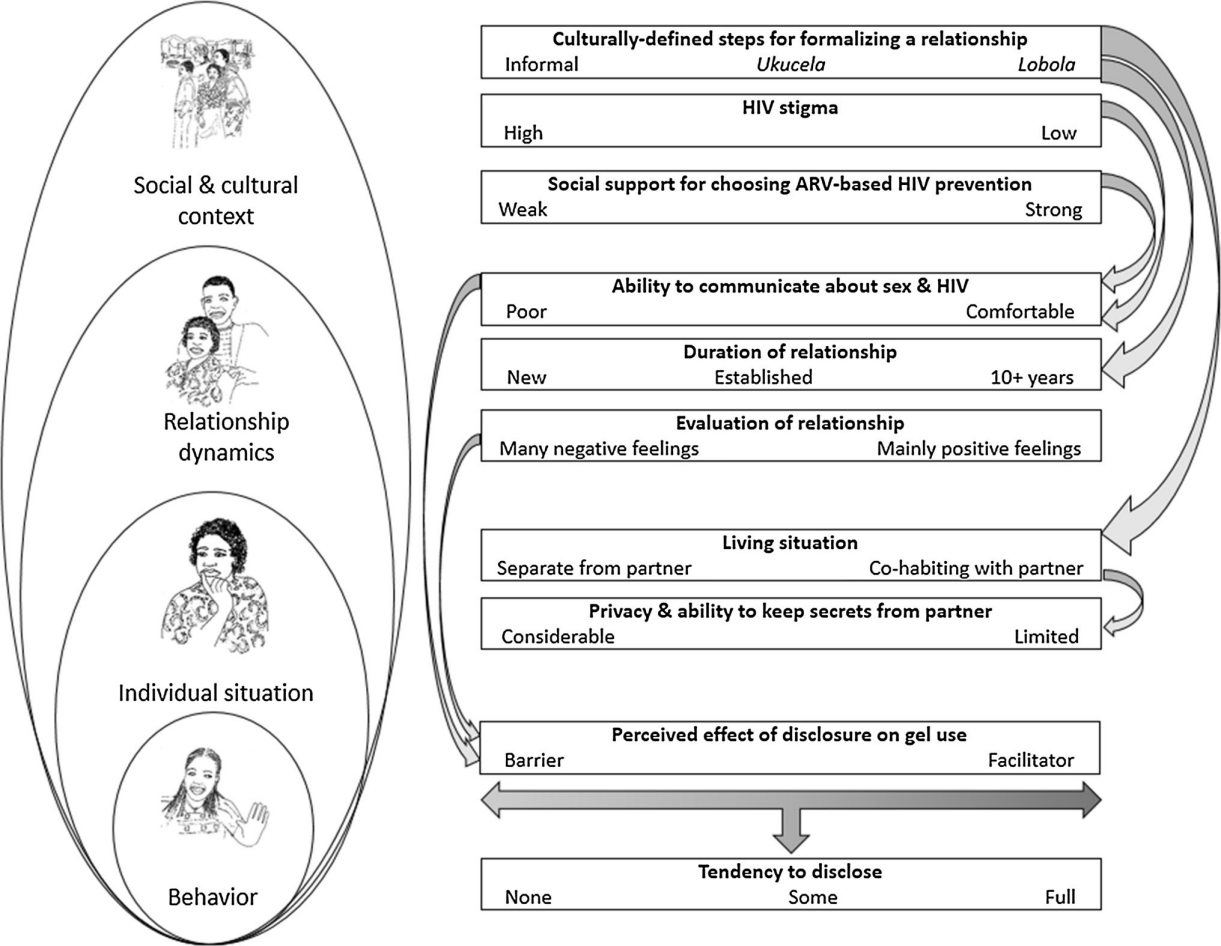
Conceptual overview of social context, gender dynamics and disclosure of ARV-based gel use in an open-label study in KwaZulu-Natal. Disclosure is a behavior enacted along a continuum from none to full. Disclosure is influenced by factors at multiple levels: the woman’s individual situation, her relationship dynamics, and the social and cultural context. These various factors, in turn, operate along continuums that push women toward greater or less disclosure. In the figure, the* right end* of each continuum pushes toward full disclosure while the* left end* pushes toward no disclosure. Factors also influence each other across levels (indicated by *curved arrows*). For example, the degree of HIV stigma in a woman’s social context influences her ability to communicate about sex and HIV within her relationship, which in turn influences the extent to which she perceives disclosure to be a barrier to or facilitator of her ability to use the gel. Whether and how much a woman discloses about gel use to her partner reflects these combined effects and can change if the factors also change

Figure 1 provides a conceptual overview of how study participants described the intersection of social context, gender dynamics and disclosure of ARV-based gel use. The factors outlined in the conceptual overview do not operate in isolation from each other. Here we first outline core components of the Zulu social context and relationship dynamics described by the participants, followed by an analysis of the social dynamics of gel use and disclosure.

### Social Context and Relationship Dynamics in KwaZulu-Natal

Almost all of the women participating in the study reported having a single male partner, but few lived with a partner and very few were married. The women in the study were not unusual in this regard, but rather their lives reflected a particular cultural setting with implications for how sexual behavior was enacted. Cohabitation (or lack thereof) may impact a couple’s opportunities for planned versus spontaneous sex, which in turn can impact a woman’s ability to use a product like tenofovir gel or to keep its use a secret. For women in the study, cohabitation was regulated by cultural expectations that the relationship would be formalized in specific ways. In Zulu culture the path to marriage begins with a practice called *ukucela* where the man sends delegates to the woman’s family to ask for good relations between their two families. During ukucela the families begin negotiations for *lobola*, a form of bride price or bridewealth paid by a man to a woman’s family before they are given permission by the woman’s family to get married. Next the woman’s family (primarily the men) write up a list of demands that the intended groom must buy and give to them, to show that he is able to take care of the intended bride—a process called *ukwembesa*. One female participant stated that all these practices, which are costly to the male partner, are done because so much has been invested in her and once she is married she no longer takes care of her family but instead takes care of his; thus her family is making up for the “loss”.

A man may be allowed to stay over when visiting a woman’s home after ukucela, and a woman may move in with her intended husband after lobola is paid but before the marriage is formalized. The practice of ukucela and visiting is more common due to the cost of marriages and lobola. The couple enjoys the privilege of being allowed to see each other when and how they please but the woman still lives with her parents. She can see her boyfriend more freely and opportunities for sex are more predictable.

Sex was generally perceived to happen spontaneously, depending on the mood of a woman or her partner, but most women also described situations when sex could be predictable. Among women who did not live with their partner, getting together, by default, tended to require more planning and women described inserting the gel under the assumption that sex would happen upon getting together. Several women said they could often tell when their partner would be in the mood for sex. Many women in this situation reported being able to prepare by inserting the gel a couple of hours ahead of time, with less likelihood of wasting gel because they miscalculated the probability of sex.

Most participants said that both men and women can initiate sex but that it was typically something men did because of a more active libido (a continual desire to have sex) and cultural norms related to men being “in charge” in the household and in the bedroom.

“There are times when a woman will just miss you and she will want it badly. And most of the time it will be that I haven’t had sex in a long time as well. Most of the time it’s usually me who initiates, I am the one who really wants it at the time.” (Urban male partner).

More than half of the women interviewed said that their partners communicated sexual desire overtly, through physical touching or direct asking; less frequently they said desire was communicated indirectly, for example, some women mentioned seeing “signs” that her partner was interested in having sex or she noticed subtle changes in his behavior (e.g., acting more kind or “sweet” to her than usual).

“He becomes extremely, extremely nice. He is a nice person naturally but then he just overdoes it so I know that tonight it’s on.” (Partially disclosed urban CAPRISA 008 participant).

One male and two female study participants suggested that tenofovir gel use could be a potential way for women to indicate interest in sex (insertion as a way of saying she is “ready”).

### Social Dynamics of Gel Use and Disclosure

An important part of our recruitment strategy was to identify women who had fully disclosed to their partners about trial participation and use of an ARV-based microbicide to prevent HIV infection. In reality, CAPRISA 008 participants described a continuum of disclosure. Among the 63 women interviewed, ten (16 %) had not disclosed any of the elements of the disclosure screening definition to their partners. Fifty-three (84 %) disclosed being in a study; 47 (75 %) also disclosed using a gel for the purpose of preventing HIV infection. Women who disclosed all three elements (n = 37, 59 %) qualified as full disclosers for the purpose of this study and were asked if they would be willing to refer their partner to participate in the study; 13 partners were successfully referred and interviewed.

Among the 10 women who did not disclose any of the screening information, six described negative feelings about their partners and four said they were unable to talk to him about HIV, about sex, or both. Half worried about their partner’s reaction if he knew they were using the gel, saying their partners may not understand the purpose. Some non-disclosers feared that their partner would disagree with her gel use and one woman worried that her partner would not allow her to use the gel. Nine women said it should be a woman’s decision to use the gel or not. In most cases women described covert gel use as an autonomous decision to potentially protect their health.

“Sometimes they don’t like the gel and when he refuse for you to use the gel on the first day it becomes difficult to talk about it the second time and its better if you just hide it from him.” (Non-disclosed urban CAPRISA 008 participant).

“…I decided to not tell him and use my thing [gel] and keep quiet… you see maybe if I came with the gel and told him that I am using this thing he would have not allowed me… Yes, maybe he would have been rude to me and maybe when we have sex he’ll complain about this-and-that, [so] I decided not to tell him.” (Non-disclosed rural CAPRISA 008 participant).

Women who partially disclosed about the gel or the study variously reported negative feelings about their partners, a general lack of communication in the relationship, no perceived need to tell, a lack of trust in their partner’s word, being in a fairly new relationship, and not being married.

“It’s just that I know him, I will not tell him. He has a loose head and I am hot headed. If I were to explain to him he would talk in a way that I wouldn’t like and then we would fight. The thing is I often say [to others] “my child, now that you are married your husband is good and you have to tell him everything.” You see, I am not married, he is not my family and I don’t trust that person even a little bit. We are together, because of what brought us together; if it ends it is over, so there shouldn’t be any of my big secrets that he knows about, that’s how I feel.” (Partially disclosed rural CAPRISA 008 participant).

Women who only disclosed study participation (n = 6) did not want to tell their partner that the true purpose of the gel was for prevention of HIV infection from a sexual partner. Some told their partner it was used for STI prevention. Women who disclosed being in a study that included use of vaginal gel for HIV prevention but did not disclose the fact that the gel contains an ARV (n = 10) were concerned about the association of ARVs with treatment for HIV infection, which could lead to a presumption that they were infected.

“I know that it won’t be as simple for him [to understand] as it is for me because I got some knowledge from CAPRISA which he doesn’t have. If I tell him it has ARVs he is going to think that it means they are just infecting us with HIV – you know, they [men] don’t understand. It is better if someone else explains it to them. So I thought it would be better if I only tell him that it prevents HIV infection, I won’t tell him what it is made up of.” (Partially disclosed urban CAPRISA 008 participant).

Partial and non-disclosers reported a higher relative frequency and wider variety of negative feelings toward their partner than fully disclosed participants. Only 7 of the 37 fully disclosed participants discussed negative feelings toward their partner, all of which were related to knowing that their partner had other sexual partners. Disclosed participants more often said that they felt comfortable discussing sensitive or important issues with their partner and none said that they felt any uncomfortableness discussing these issues with their partner.

“For using the gel I realized that I trust myself now and also that in our relationship we are able to talk about serious stuff; I can see that because we talk about these things, I am not scared to talk to him about something and he is also not scared. Using the gel made us to be able to talk about many things regarding sex.” (Disclosed rural CAPRISA 008 participant).

Full disclosers more frequently noted being with partners for more than 10 years. While some of the full and partial disclosers stated they lived with their partners, none of the non-disclosers did. Many fully disclosed women noted that they disclosed to their partners before using the gel. Full and partial disclosed participants often said they found it difficult to keep gel use hidden from their partner, and some said that they were unable to use the gel secretly due to gel perceptibility (e.g., wetness) and sharing a living space; a few also expressed concern that the gel may affect their partner in some way.

“I decided to tell him because I also want him to know that when I wake up and do something else in the house then he mustn’t be shocked and say what is this woman doing now? I want him to know that ‘ok she is using that thing of hers’.” (Disclosed rural CAPRISA 008 participant).

“When I first started I would sneak around. I did not tell him that I was coming here, until he caught me. He asked me where I go every month. I told him that I go to a clinic in Durban and I explained about the gel and … then he liked it. I then showed him because he wanted to see it.” (Disclosed urban CAPRISA 008 participant).

In general, male partner support for tenofovir gel use was a result of rather than a motivation for disclosure.

Discussions about disclosure sometimes highlighted the way women’s lives change over time, and how they needed to be aware of the potential for relationships to change when deciding what to tell their partners. Disclosure was a process for women, not an event. As a process it involved (1) evaluating the disposition of one’s partner toward HIV and clinical trial participation, (2) evaluating the sincerity or seriousness of the relationship (both of which may involve “testing the waters” by proposing a hypothetical situation with the partner), and (3) choosing what level of disclosure is appropriate given the relationship context and what the woman feels is necessary to meet her adherence and psychological needs (some women reported that they felt that they ought to disclose their product use, but not necessarily to disclose “everything”). The following quote exemplifies how this process is subject to many considerations.

“The reason why [I decided not to tell my partner], I was dating my ex-boyfriend, the father of my child and he did not want to hear about the gel because he would hear [negative] stories about it. So he thought that if you go to CAPRISA you are [HIV] positive, so he did not understand and I knew that even if I tell him but he wouldn’t understand. So we had been together for 10 years, we had a child and then we broke up. So after we broke up I stayed single until I found my current partner. My current boyfriend is so understanding because I told him that I was in a study before – I did not tell him that I am still participating – there was a study where they were testing the gel and everything and it was proven to be working. He was okay with it and said wow okay and where is that gel now. You see, I was like must I tell him or not, what if he tells me not to use it. But … I can see that if I can tell him he will not have a problem because he is very good, yes. But I will disclose to him, yes I will tell him because he is a good person and I am sure he will support me in all this thing, he is not like my ex-boyfriend.” (Non-disclosed rural CAPRISA 008 participant).

The importance of the dissemination of information about the CAPRISA 004 trial findings for partner support of gel use was also evident in statements from some of the women, indicating how the particulars of a blinded clinical trial may influence adherence. Dissemination helped to generate social support for gel use.

“In [CAPRISA] 004 we did not know much about the gel. And our partners did not know because we decided not to tell them during that time and they only knew [when] the study ended. But now we don’t have any problem, they know now, we don’t hide it from them and they support us so it [gel use] is easy now.” (Disclosed urban CAPRISA 008 participant).

“I told him that I want to help South Africa and the world so I want to participate so that if this thing is licensed one day I can say I had an input. Seriously!…He just supported me…He is a well-informed person. I think he knew, he knew about the study, he must’ve heard about it from somewhere. Maybe he heard his friends talking about this thing and he’s seen it on TV so he knows about it. He doesn’t have a problem with it.” (Partially disclosed urban CAPRISA 008 participant).

Nearly all of the female participants across all sites and discloser types said that gel use should ultimately be at a woman’s discretion, specifically because of the design of the product as a female-controlled preventative method for women. About half of the male participants agreed with this sentiment. However, there was also general consensus that women and men should discuss the use of the gel and come to some mutual agreement regarding its use. Women more often described this as the ideal situation, while men typically described this as a means of avoiding conflict should he find out about her use of the gel. In situations where discussion isn’t practical or where the man disagrees with his partner’s use of the gel, several women (n = 9) and one male partner felt that she should choose to continue to use the product in secret. Only one male participant, from the rural site, said that it should be a man’s decision to use the gel, on the basis that men can more easily control the course of sex acts once sex is initiated.

“I think it should be a shared decision but sometimes you get those Zulu men who want you to do as they say and they do not want to use things that they do not know of like the gel; in that case a woman should decide.” (Partially disclosed urban CAPRISA 008 participant).

“I think that if both persons know of it and [are] educated about it, they both should have a right to talk about it. […] It would be dangerous if [it] happens that the one finds out because you can’t conceal something forever. If he catches you, and to me that is similar to one concealing a secret lover. You will find that they will quarrel and [there] will be a fight that ensues and they may eventually hurt each other or break up. Whereas if they both know what is going on, I doubt that there would be a problem.” (Rural male partner).

## Discussion

In this study we sought to gain a nuanced understanding of product use in the context of an open-label implementation trial (CAPRISA 008) of an ARV-based microbicide with some evidence of effectiveness (tenofovir gel). We explored the relationship between partner disclosure and women’s experiences related to product use. The women in this study had previously participated in a blinded, placebo-controlled, randomized trial of tenofovir gel (CAPRISA 004); quantitative and qualitative data collected from participants in the CAPRISA 004 trial indicated that disclosure influenced adherence though no measureable impact on product effectiveness was identified [6, 7].

As in the earlier CAPRISA 004 trial, women in the open-label CAPRISA 008 implementation trial described a continuum of disclosure that highlights the intersection of women’s autonomy, gender dynamics within couples, and HIV stigma. The problems and challenges of disclosure were reflected in the levels of disclosure and highlighted the fact that disclosure was a process rather than an event. Similar processes have been noted for other microbicide trials and are likely to be an important consideration in the roll out of any effective microbicide-type product [10]. Both women and men understood that before a woman could discuss use of tenofovir gel with a man, the couple must be able to communicate with each other on sensitive issues like sex and HIV. Lack of open communication on these issues meant a woman could not negotiate introducing any method of HIV prevention in the relationship. Thus the ability of a couple to communicate openly about sex and HIV largely determined whether a woman would consider covert use of the gel, and how people other than her partner would likely judge such use.

Despite such generalities of process, the way in which gender dynamics are addressed and negotiated in product marketing and distribution will need to reflect the specifics of social context. The negotiation process for women participating in the CAPRISA 008 implementation trial was determined in part by relationship dynamics including the duration of the relationship, the living situation, and an evaluation of the relationship (e.g., partner intimacy and relationship expectations). Culturally-defined steps for formalizing the relationship, in turn, influenced the relationship dynamics. This is a quite different context than, for example, the situation of Luo widows in Kenya who are expected to engage in sexual intercourse to remove impurity ascribed to a woman after her husband’s death as well as preceding specific agricultural activities, building homes, funerals, weddings, and other significant cultural and social events [11]. Gender dynamics may differ significantly between populations yet shape HIV risk and prevention outcomes in similar ways [12].

Importantly, the degree to which gel use was disclosed (if at all) by CAPRISA 008 participants was held within the user’s prerogative. Disclosure clearly facilitated adherence for many women. But this did not diminish the fact that others reported using the gel effectively in the absence of any disclosure, while in some situations disclosure was itself a barrier to adherence. Given the potential for disclosure to be both a barrier and facilitator, depending on specific details of a woman’s relationship with a male partner, women must have opportunity to use these technologies without their partners’ knowledge or acquiescence. Women who cannot openly negotiate HIV prevention with their partners are precisely the women most in need of effective products designed and packaged in ways that facilitate covert use in the contexts of their daily lives. The flexibility with regard to timing of tenofovir gel insertion relative to the timing of sex was a notable benefit in this regard, especially for women concerned about their partners’ perceptions of gel (e.g., coldness, wetness) and interpretations of those perceptions (e.g., infidelity).

The piece of information that women were least likely to disclose to their partners was the fact that the active ingredient of the gel is tenofovir, which is an ARV. This was mainly due to the prevalent (and accurate) understanding of ARVs as treatment for HIV infection and the social stigma surrounding HIV. Many women saw no need to say anything about tenofovir gel containing an ARV, and the gel did not look like any ARV used for treatment. An analysis of local perspectives on using ARV as pre-exposure prophylaxis to prevent HIV in the MTN 003 (VOICE) trial in Johannesburg, South Africa revealed similar social meanings of ARVs and also highlighted the fact that ARV pills were often recognized as HIV treatment medications while tenofovir gel lacked this automatic association [13]. The CAPRISA 008 participants expressed a nuanced understanding about the presence of the ARV tenofovir in the gel and the fact that it was there with the intent to prevent HIV infection. Nonetheless, they were often challenged in their attempts to explain this nuance to others, including their male partners.

Dissemination of CAPRISA 004 research findings in the local community and support from CAPRISA counselors were often cited by women in this study as helpful for gaining confidence in their personal acceptance of tenofovir gel and in winning the support of their partners and others for their use of the gel. These findings suggest that the social and cultural context of stigma surrounding ARVs should not be presumed to be negatively fixed with reference to the use of ARVs for prevention. Open discussion and dissemination of information about ARV-based prevention options in the clinic setting and in local trusted media can help create a supportive social context for women choosing to use ARV-based methods.

Several limitations should be noted. The findings from this study are descriptive and are not intended to prove or disprove causal relationships with regard to adherence. They are meant to inform how we think about and can work with women, their male partners, and local communities to support uptake and use of biomedical HIV prevention products such as tenofovir gel. The number of male participants was small and, of necessity, limited to those men who were fully informed about their partner’s use of an ARV-based gel for HIV prevention in the context of an open-label implementation trial. We successfully enrolled 35 % of eligible male partners; non-participation reflected several reasons including women who did not want to refer partners despite full disclosure, the challenges of enrolling men who were labor migrants elsewhere, and men who were not interested. Though limited, the partner data provided valuable insights given the minimal amount of data currently available from male partners of women enrolled in ARV-based prevention trials. Innovative approaches to enrolling male partners are needed. We did not collect data on adherence as part of this study, rather, our emphasis was on understanding the experience of tenofovir gel use among women and some of their male partners. Once the adherence results are available from the CAPRISA 008 trial, the findings of this analysis (and others underway from additional CAPRISA 106 data) will contribute to our understanding of the trial results.

Our findings answer a number of important questions about microbicide use and gender dynamics in the context of an open-label trial, and point to others in need of further research. We described the important role of the cultural context of sexual relationships, which highlights the need for future studies to look more closely at the way cultural particulars may drive product effectiveness through impacts on behavior. A focus on individual behavior and decision-making about product use in the absence of contextual understanding may lead to misguided assumptions about what women need to make a product “work” in their lives. Our findings confirm the generalizability of the importance of gender dynamics for women’s negotiation of HIV prevention product use beyond the context of the randomized, blinded, placebo-controlled trial. However, we also saw that although women in this study could reference evidence of safety and effectiveness of tenofovir gel, it did not eliminate or minimize all of the gender and social barriers to product use. We need a clearer understanding of the cultural positioning of biomedical HIV prevention products, if we are to see their promise fulfilled. Although a supportive social context is a facilitator for adherence and may be a result of (rather than a motivator for) disclosure, data collected through this study show that microbicides remain an important HIV prevention method and a lack of disclosure does not necessarily inhibit their use. As we continue towards the goal of zero new HIV infections, development of women-controlled methods remains imperative.
